# Repetition positivity following auditory intensity or frequency changes in young normal-hearing adults

**DOI:** 10.3389/fnins.2025.1679647

**Published:** 2026-01-16

**Authors:** Büşra Altın, Hasan Colak, Charlie Maskery, Kai Alter, William Sedley

**Affiliations:** 1 Department of Audiology, Hacettepe University, Ankara, Türkiye; 2 Biosciences Institute, Newcastle University, Newcastle upon Tyne, United Kingdom; 3 Faculty of Medical Sciences, Translational and Clinical Research Institute, Newcastle University, Newcastle upon Tyne, United Kingdom; 4 Faculty of Modern and Medieval Languages and Linguistics and the Languages Sciences Interdisciplinary Research Centre, University of Cambridge, Cambridge, United Kingdom

**Keywords:** habituation, mismatch negativity, predictive coding, repetition positivity, repetition suppression

## Abstract

**Introduction:**

Generative mechanisms of perception such as predictive coding are used to explain how the brain perceives the world; such mechanisms are often experimentally probed using “deviant” stimuli that violate established patterns (including mismatch negativity), which also elicit responses related to lower-level processes such as stimulus-specific adaptation. However, little is still known about brain responses that indicate the strength of sensory predictions or reinforcement of sensory representations. Repetition positivity (RP) is a positive polarity evoked potential that gradually increases with each repetition of a stimulus, and is thought to reflect progressive strengthening of auditory sensory memory and/or habituation to repetitive stimuli. The aim of this study was to compare RP that follows a change in stimulus frequency with that following a change in stimulus intensity, the latter having not previously been studied.

**Methods:**

We used roving sequences of isochronous 5 kHz pure tones (300 ms duration, 300ms inter-stimulus interval), which changed in frequency by 1 kHz (Experiment 1) or in intensity by 12 dB (Experiment 2) after every 30 stimuli. All changes were roving, such that an increase would be followed by a decrease, and vice versa.

**Results:**

Event-related potentials recorded with EEG indicated that frequency changes in either direction were followed by RP, whilst only intensity increases were followed by RP, and only a weak visual trend toward RP was apparent for intensity decreases. Observed RP was best explained by a logarithmic function over successive stimuli.

**Conclusions:**

RP robustly follows increases, but not necessarily decreases, in stimulus intensity, which appears smaller in amplitude than that elicited by similarly salient frequency changes, and reaches a plateau sooner. These observations offer insight into how intensity is processed similarly yet differently to other sensory attributes in an adaptive or predictive coding framework, and might have future utility in the study of clinical conditions related to aberrant predictive mechanisms.

## Introduction

To perceive the world, the brain predicts current and upcoming sensory inputs by constructing internal models of the external environment, with one such formulation being predictive coding. In this model, representations of the causes of sensory input are optimized by minimizing prediction errors (discrepancies between prediction and ascending sensory input) within the framework of a hierarchical model (Friston and Kiebel, 2009). In generating prediction error, predictions are transmitted to lower levels of a cortical sensory hierarchy (or to the lowest-level sensory inputs) where they are compared with lower-level representations (or to the sensory input). The prediction error is then sent back up through forward connections to improve the predictions and thus reduce prediction error. This model provides a theoretical basis for both visual and auditory perception (Kumar et al., 2011), as well as potentially all other sensory modalities. However, many features of stimulus responses can also be explained on the basis of local adaptation to particular sensory inputs.

Specific brain responses that are widely measured provide a quantification of how much a given stimulus concords with or violates existing predictions or representations. Event-related potentials (ERP), which reflect stimulus time-locked activity of large-scale neuronal populations, are one such category of objective measure. Research has demonstrated that when a stimulus is immediately preceded by multiple repetitions of the same stimulus, a sensory memory trace is created. It is contended that stimulus predictability facilitates the formation of a probabilistic anticipation of future stimuli, which is what drive these repetition effects (Baldeweg, 2007; Grotheer and Kovács, 2016).

Repetition Positivity (RP) is an ERP component that has been identified as a neurological correlate of sensory memory trace development. RP is observed as a positive wave in ERPs that grows with the recurrence of auditory stimuli. It is characterized by repetitive suppression and amplification effects that occur in various auditory components between 50 and 250 ms following sound onset (Recasens et al., 2015; Ylinen and Huotilainen, 2007). The N1 peak involves contributions from primary and non-primary auditory cortical regions, and centers on planum temporale (posterior to primary auditory cortex). The P2 response and RP are likewise distributed across multiple auditory areas. Also, RP includes contributions across some non-auditory areas. The later timeframe of RP than N1 suggests mechanisms involved in later stages of processing (Baldeweg, 2007; Näätänen and Picton, 1987; Nelken and Ulanovsky, 2007).

RP potentially includes mechanisms within predictive coding (RP as top-down suppression of predicted stimuli) and stimulus-specific adaptation (SSA) frameworks, suggesting both top-down suppression and local adaptation might contribute (Baldeweg, 2007; Nelken and Ulanovsky, 2007). So, it could be interpreted as a marker of reduced prediction error (suppression) when input matches a learned model, complementary to MMN as the error signal to deviants; it is also aligned with SSA in auditory cortex. The coexistence of frontal and mastoid RP supports contributions of both top-down model-based suppression and local adaptation mechanisms, so it is thought that a candidate mechanism for auditory trace formation. It might reflect the strengthening of an auditory sensory-memory trace and adaptation of reaction to standard stimuli. Furthermore, it is closely related to the frontal cortex's modulation of this adaptive response in the context of expectation and error prediction (Baldeweg, 2007; Garrido et al., 2009; Cooper et al., 2013).

Repetition Positivity rises with the number of repetitions of a standard stimulus in a roving-stimulus paradigm. The augmentation of RP is believed to indicate the encoding of repeated stimulus properties such as intensity and frequency in the auditory cortex (Recasens et al., 2015; Cooper et al., 2013; Haenschel et al., 2005). The brain processes sound intensity and frequency through complex mechanisms involving both peripheral and central auditory pathways. In peripheral mechanisms, the cochlea decomposes sound into its frequency components, and with the auditory nerve encodes the intensity of these components and transmits information to the brain with the auditory pathways (Kohrman et al., 2021; Tao et al., 2017). The cochlea and central auditory system maintain a tonotopic map, where different frequencies are represented spatially. This organization is preserved at all levels of the auditory pathway, including the IC and auditory cortex (Davis, 2005). Conversely, no such topic intensity mapping is present.

As sound information ascends through the auditory pathway, it reaches various brain regions, including the inferior colliculus (IC) and auditory cortex. Neurons in these areas respond selectively to sound intensity and frequency (Davis, 2005; Morrison et al., 2014). Functional MRI studies have shown that brain activation in the auditory cortex is more closely related to perceived loudness rather than physical sound pressure level, indicating a transformation from physical intensity to perceptual loudness (Behler and Uppenkamp, 2016; Uppenkamp and Rohl, 2011).

Changes in a stimulus following an established repetitive pattern (i.e., deviant stimuli) elicit characteristic brain responses, including a negative scalp potential termed mismatch negativity (MMN; Garrido et al., 2009; Näätänen, 1995) and increased firing of certain deviant-responsive neurons at all studied levels of the auditory pathway (Carbajal and Malmierca, 2018). Changes in acoustic stimulus frequency are the most studied type of deviant, but changes in any one of a wide range of stimulus features in various sensory modalities elicit MMN. Stimulus changes producing MMN are also associated with increased firing rates of specific neurons at multiple levels of the auditory pathway. Interestingly, MMN is elicited by deviants that either increase or decrease in stimulus intensity, whereas single neurons responsive to intensity changes have only been demonstrated for intensity increases, not decreases (Carbajal and Malmierca, 2018).

In this study, we aimed to investigate whether, and how, RP is elicited following changes in sound intensity, and how this compares to the well-established phenomenon of RP following changes in frequency. Given that there are clear distinctions between the occurrence of deviant responses to upward vs. downward changes in stimulus intensity, we also aimed to compare RP between the two directions of stimulus change.

## Materials and methods

The study took place in the Auditory Cognition Lab, Newcastle University, in a dedicated soundproof facility. The research was approved by the Newcastle University ethical review process (ref 28762/2022), all participants provided prior informed written consent, and all research was conducted in accordance with the Declaration of Helsinki. The consent form includes the information about EEG test, RP, and audiometry, and the inclusion and exclusion criteria of the study.

### Subjects

Two groups of healthy adults (aged 18–35) with normal hearing participated in the study, which comprised two experiments. Experiment 1 featured changes in stimulus frequency, and included 14 subjects with ages of mean 21 and SD 0.5 years (3 female). Experiment 2 featured changes in stimulus intensity, and included 17 subjects with ages of mean 28 years and SD 0.5 years (6 female). Participants were recruited from affiliated volunteer lists at Newcastle University. Subjects with history of neurological or psychiatric disorders or hearing difficulties were excluded from the study.

Pure-tone audiometry was performed for all participants, and participants only included with a normal audiogram, which we defined as < 25 dB HL for all the audiometric frequencies between 0.25 and 8 kHz. We did not need to exclude any subjects based on this criterion.

### Experimental procedure

The two experiments were identical in most respects, including all of the following. They involved a passive listening task, during which subjects watched silent subtitled movie of their choosing. Stimuli comprised sequences of isochronous pure tones of 300 ms duration, 10 ms onset/offset ramps, and 300 ms inter-stimulus interval, which roved in one stimulus attribute every 30 stimuli, with the direction of roving alternating, such that blocks of 30 successive stimuli alternated between just two values. The starting value was randomized. A total of 6,000 stimuli were presented, i.e., 3,000 per stimulus value, or 100 per value per sequence position. Stimuli were presented diotically via HDA200 headphones (Sennheiser, Wedemark, Germany)

In Experiment 1, stimulus frequency roved between 5 and 6 kHz, and intensity was fixed at 77 dB HL. In Experiment 2, stimulus intensity roved between 65 and 77 dB HL, and frequency was fixed at 5 kHz. Informal piloting within the research team found this intensity difference to be similarly salient to the 1 kHz frequency difference in Experiment 1. Electroencephalography (EEG) data were recorded using a 64 channel Biosemi Active two system (Biosemi Inc.) at 256 Hz.

### EEG data preprocessing

In EEGlab (Delorme and Makeig, 2004), EEG data were re-referenced offline to a P9/P10 reference, approximating to linked mastoids. Data were bandpass filtered using a non-phase-distorting filter between 1 and 30 Hz. If needed, based on visual inspection, bad channels were interpolated. ICA was performed using the “runica” function, and components containing predominantly ocular artifacts were removed. Periods of continuous data containing artifact were determined using the “clean_rawdata” function with default settings. Data were then epoched between −100 and 500 ms peristimulus time, baseline corrected to −100 to 0 ms, and data from FCz formed the basis of all subsequent analysis, given that this montage is well-suited to revealing auditory sources, and is expected to yield only positive RP changes. Epochs were averaged within each combination of sequence position and frequency/intensity.

### Statistical analysis

Statistical analysis was performed using custom-written Matlab (the Mathworks inc.) code. Because we had a strong hypothesis about progressive positive shifts in ERPs over successive stimuli, and were not seeking negative changes, and in light of the relatively low number of stimuli per sequence position, we used one-tailed statistics, as described below (no changes were found between one and two-tailed permutation tests). Furthermore, we limited the statistically analyzed time window to 100–300 ms post-stimulus, as we only expected RP to occur within this timeframe.

Analysis was based on a non-parametric permutation approach, seeking significant time points, rather than clusters. In each of 1,000 permutations, we randomized the labeling of the stimulus frequencies/intensities and sequence positions; analysis for the unpermuted data was the same but without this randomization. To make better use of all trials, thereby reducing noise in the data, we calculated a linear regression coefficient for each time point, with amplitude as the dependent variable, and stimulus sequence position the independent variable. This was performed between sequence positions 2 and 30, to avoid incorporating MMN responses. Regression coefficients were multiplied by 29, to reflect the full degree of modeled change across the whole stimulus sequence. A null distribution was created by taking the largest positive regression coefficient within 100–300 ms for each permutation. The 50th-highest value in the null distribution was taken as the significance threshold for *p* < 0.05 (one-tailed), and all time points within the 100–300 ms range with regression coefficients for the unpermuted data that exceeded this value were deemed to show significant RP.

Where significant RP was identified, we sought to characterize its time course over successive stimuli. For each sequence position, a grand average response was calculated as the mean across all subjects and all time points showing significant RP. Linear, logarithmic and cubic functions were fitted in each instance, and adjusted R squared values compared in order to determine the best fit, after applying a penalty for model complexity.

## Results

**Figure 1:**
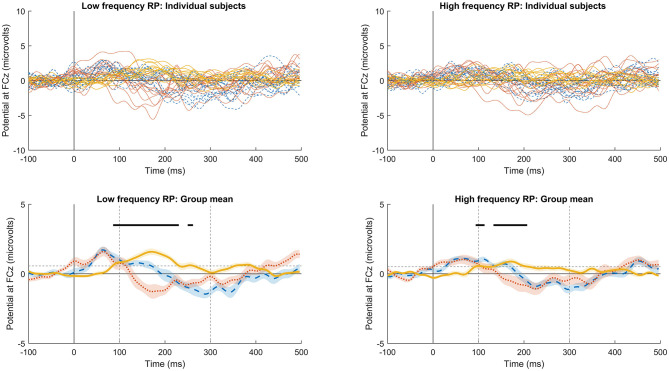
Individual subject and group-significant RP following frequency changes. Upper plots show individual subject data, and lower plots the group mean and statistical testing results. Left plots indicate the low-frequency (5 kHz) responses, and right the high frequency (6 kHz). Dotted red lines indicate stimulus 2 in the sequence, dashed blue lines stimulus 30, and solid orange lines indicate RP calculated as the scaled linear regression coefficients across successive stimuli. Dashed gray straight lines indicate the limits of the data space used for statistical testing, with vertical lines indicating the statistically analyzed time window, and horizontal lines the threshold for one-tailed statistical significance. Solid black lines indicate time points with significant RP. Areas marked as significant outside of the vertical dashed lines must be interpreted with caution, as these time points were not included in setting significance thresholds.

**Figure 2:**
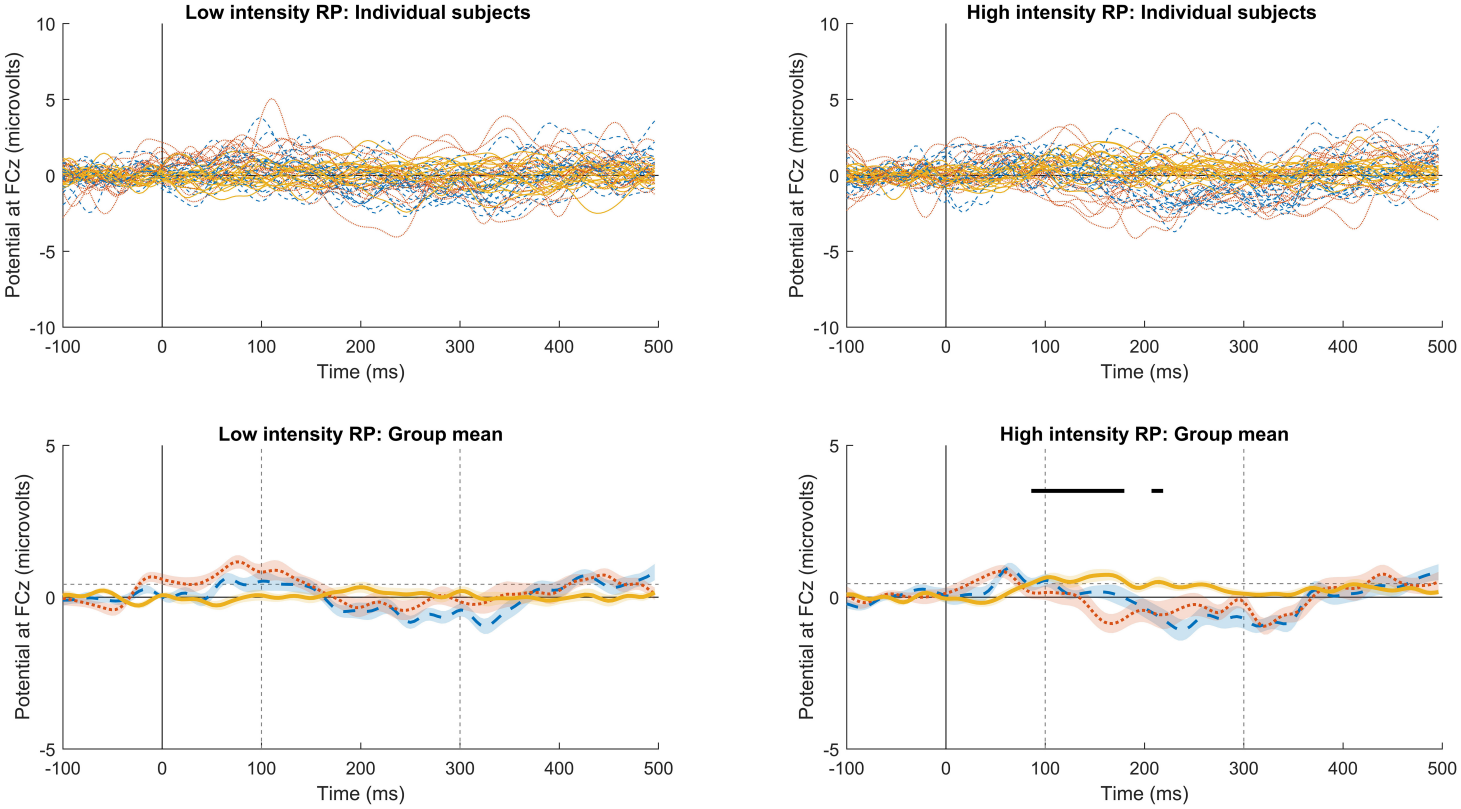
Individual subject and group-significant RP following intensity changes. Upper plots show individual subject data, and lower plots the group mean and statistical testing results. Left plots indicate the low intensity (65 dB HL) responses, and right the high intensity (77 dB HL). Dotted red lines indicate stimulus 2 in the sequence, dashed blue lines stimulus 30, and solid orange lines indicate RP calculated as the scaled linear regression coefficients across successive stimuli. Dashed gray straight lines indicate the limits of the data space used for statistical testing, with vertical lines indicating the statistically analyzed time window, and horizontal lines the threshold for one-tailed statistical significance. Solid black lines indicate time points with significant RP. Areas marked as significant outside of the vertical dashed lines must be interpreted with caution, as these time points were not included in setting significance thresholds.

**Figure 3:**
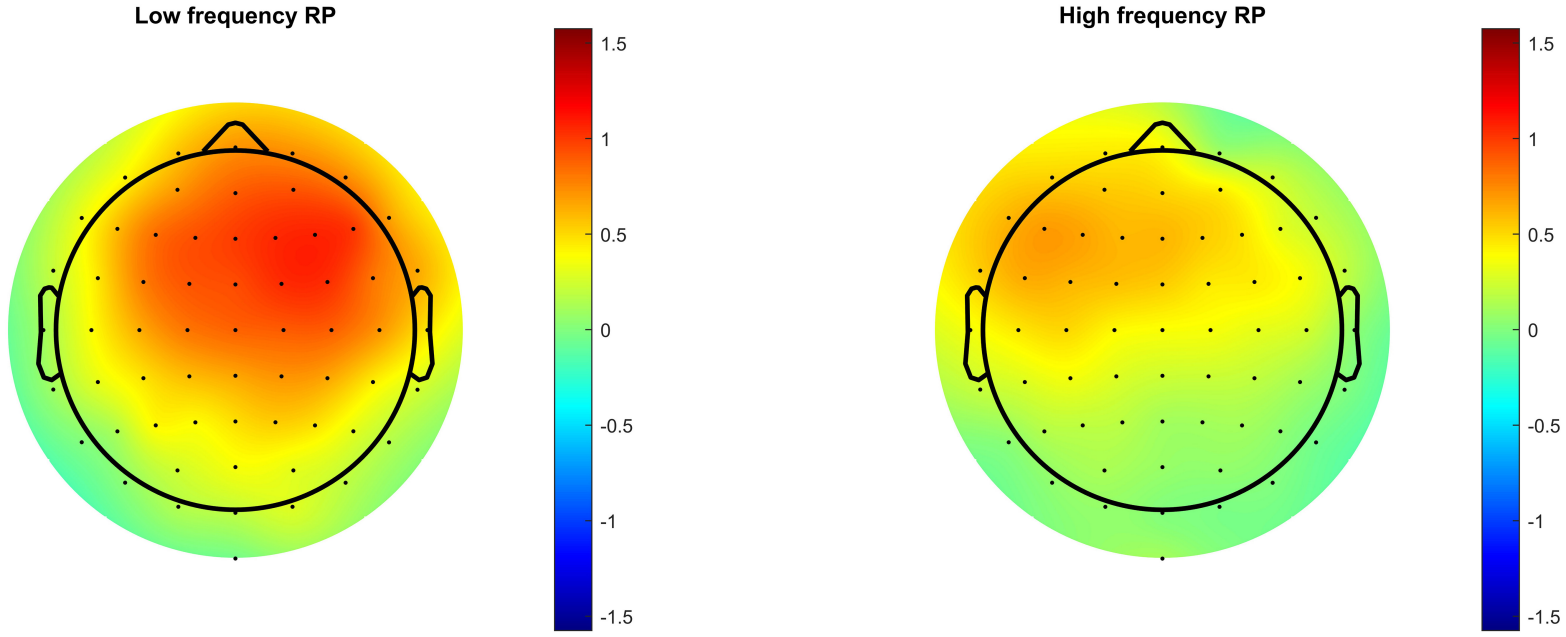
Scalp topographies of RP following stimulus frequency change.

**Figure 4:**
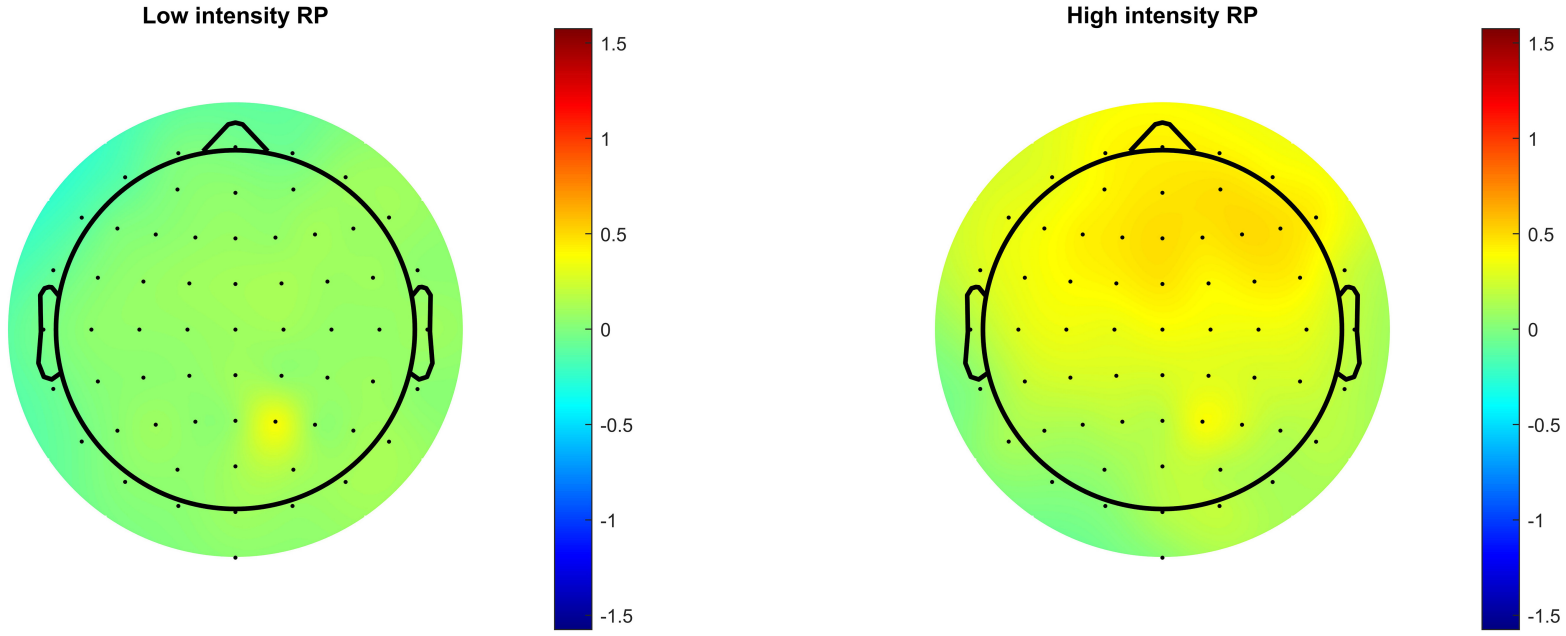
Scalp topographies of RP following stimulus intensity change.

Following frequency changes (Figure 1), significant RP was observed between 100 and 230 ms, peaking at 172 ms, for low-frequency stimuli, and between 100 and 207 ms, peaking at 168 ms, for high frequency. It is possible that significant RP occurred from as early as 86 ms for low frequency, and 94 ms for high frequency, but these time points before 100 ms were not part of the pre-defined time window of analysis. Following intensity changes (Figure 2), significant RP was not observed for low intensity stimuli, though a non-significant trend was observed around 200 ms. For high intensity stimuli, significant RP was observed between 100 and 180 ms, peaking at 164 ms. RP might have begun as early as 86 ms, but this was outside the time window of analysis. The spatial distribution of the RP can be seen via scalp topographies of the frequency (Figure 3) and intensity changes (Figure 4).

**Figure 5:**
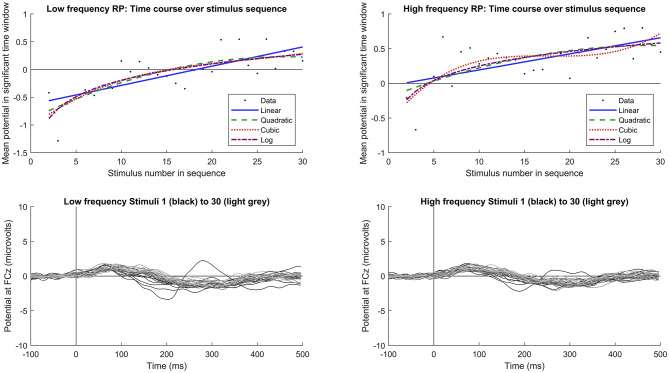
Time course of RP following stimulus frequency change. Upper plots show the grand average response for each stimulus sequence position (starting from 2, to avoid deviant responses) across subjects and peristimulus time points showing significant RP (black dots), along with fitted linear (blue), quadratic (dashed green), cubic (dotted red), and logarithmic (dashed purple) functions. Lower plots show the mean response to each stimulus sequence position from 1 (deviant, black) to 30 (lightest gray), denoted by progressively lightening shade. An MMN response would typically be calculated by subtracting either the last standard (lightest gray) or mean of all standards from the deviant stimulus (black). Left plots indicate responses to low frequency stimuli, and right to high frequency.

**Figure 6:**
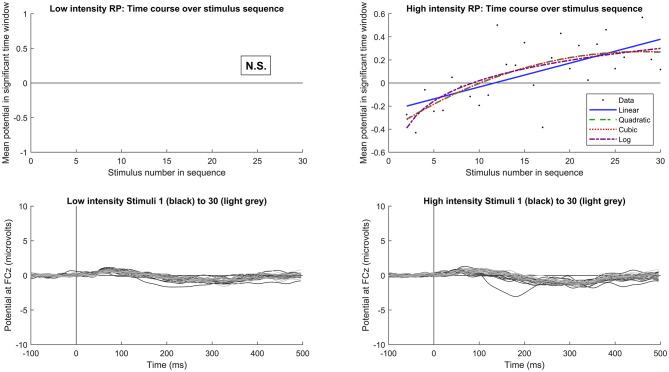
Time course of RP following stimulus intensity change. Upper plots show the grand average response for each stimulus sequence position (starting from 2, to avoid deviant responses) across subjects and peristimulus time points showing significant RP (black dots), along with fitted linear (blue), quadratic (dashed green), cubic (dotted red), and logarithmic (dashed purple) functions. No data are presented for low intensity stimuli, as significant RP was not observed. Lower plots show the mean response to each stimulus sequence position from 1 (deviant, black) to 30 (lightest gray), denoted by progressively lightening shade. An MMN response would typically be calculated by subtracting either the last standard (lightest gray) or mean of all standards from the deviant stimulus (black). Left plots indicate responses to low intensity stimuli, and right to high intensity.

For the time course of RP across stimulus sequence positions, a logarithmic fit outperformed the other models, showing the highest adjusted R-squared values for both frequency increase and decrease, as well as for intensity increases. This suggests that RP changes rapidly at the beginning of the sequence and then stabilizes after the first few presentations. Adjusted R-squared values for all model fits across conditions are summarized in Table 1. Based on visual inspection of the model fits, RP following frequency changes (Figure 5) reached a plateau at around the 30th stimulus. Conversely, RP following intensity increases (Figure 6) reached a plateau at around the 20th stimulus. However, we are cautious in placing too much emphasis on these results, as they were not determined via any formal statistical process.

**Table 1:** Adjusted R-squared values, for competing model fits, for the time course of RP across the stimulus sequence.

**Conditions**	**Linear**	**Quadratic**	**Cubic**	**Logarithmic**
Freq. dec.	0.54	0.58	0.57	0.59
Freq. inc.	0.37	0.38	0.44	0.46
Int. dec.	N/A	N/A	N/A	N/A
Int inc.	0.41	0.44	0.41	0.45

Freq., frequency; Int., intensity; dec., decrease; inc., increase.

## Discussion

### Summary of results

In this study, we have demonstrated that repetition positivity, which appears as a gradually increasing positive deflection across successive repeated stimuli, follows changes in frequency in either direction, but only necessarily following increases in stimulus intensity, rather than decreases. RP was evident from around 100 ms, or possibly as early as 86 ms, and persisted until 230 ms at the latest. It was best explained by a logarithmic function for both frequency and intensity changes, reaching a plateau at around 20 stimuli for intensity and around 30 stimuli for frequency.

The auditory system's response to changes in intensity and frequency is shaped by processes at multiple levels, from the cochlea to the cortex. At the peripheral level, basilar membrane mechanics and afferent neural firing determine how well intensity and frequency can be resolved. At more central levels, adaptive and predictive mechanisms such as stimulus specific adaptation (SSA), repetition positivity (RP), and mismatch negativity (MMN) support the detection of changes over time. These cortical mechanisms interact with top down prediction processes, which influence how strongly the system adapts to repeated sounds and how sensitive it is to new or unexpected events. Through this network, the auditory system is able to maintain continuity and detect change with high temporal and spectral precision (Haenschel et al., 2005; Costa-Faidella et al., 2011a).

### Interpretation of RP for intensity changes, but only increases

Within generative frameworks of perception such as predictive coding, repeated auditory stimuli lead to increasingly precise predictions, resulting in neural responses that become progressively more “tuned” to the expected input (Bader et al., 2017). If this is the case, then our results indicate the possibility that RP is unlikely to be directly reflecting precision of the predicted intensity or loudness, as changes in intensity were just as strong in each direction, yet only increases led to RP occurring. RP can also be explained in the framework of stimulus-specific adaptation, in which intensity decreases are not necessarily equivalent to increases. Under adaptation, repeated stimulation drives neurons into a reduced-response state. An increase in intensity represents a stronger deviation from this adapted state and therefore produces a clear release from adaptation, resulting in an identifiable RP response. However, an equivalent decrease in intensity moves the stimulus further into a lower-energy range where neurons are already partially adapted which may be insufficient to generate an RP (Haenschel et al., 2005; Costa-Faidella et al., 2011b; Baldeweg, 2006).

Early investigations demonstrated that the auditory system rapidly extracts patterns from sequential sound presentations, as evidenced by ERP components such as mismatch negativity (MMN) and P3a. Concurrently, increases in repetition positivity are thought to reflect a parallel process of sensory memory trace consolidation (Bendixen et al., 2012). The gradual enhancement of the RP with each repeated stimulus is interpreted as evidence of a mechanism involving stimulus-specific adaptation and synaptic plasticity, whereby the memory trace is propagated from higher-order processing regions back to early sensory cortices (and wherein top-down signals from higher-order auditory and prefrontal regions enhance the activity in primary auditory cortex; Garrido et al., 2009). Based on our results, such an explanation might need to specifically refer to stimulus-specific adaptation to topically represented features such as frequency, and as opposed to other forms of adaptation applicable to intensity such as firing rate adaptation or dynamic range adaptation.

Auditory repetition positivity does not exist in isolation but rather as part of a constellation of ERP responses that together provide a more comprehensive picture of auditory processing. Previous work has shown that the P2 component and related positive deflections, including RP, reflect processes such as memory trace strengthening and auditory object formation (Recasens et al., 2015; Bader et al., 2017; Norena et al., 1999). Importantly, these components exhibit clear intensity dependence. MMN is one of the most extensively studied components in this context. While MMN reflects the detection of deviations from an established auditory regularity, its amplitude is positively related to repetition positivity, particularly if the response to the last standard in a sequence (which has already been subject to RP) is subtracted from the deviant response to calculate MMN. As well as this methodological relationship, it is also possible that as the internal memory trace becomes stronger (as indexed by more prominent RP), the response to a deviant increases in amplitude, reflecting violating a more precise prediction (Costa-Faidella et al., 2011a; Yukhnovich et al., 2023). The stronger MMN responses for frequency deviants than for intensity deviants (Näätänen et al., 1993; O'Reilly, 2021). This is in line with our study, which show a larger RP effect with frequency. Similarly, the P3a—which is associated with the reorientation of attention toward deviant or unexpected stimuli—can be modulated by the prior repetition of standards, implying that robust memory trace formation (indexed by RP) influences later stages of cognitive evaluation.

Temporal regularity is another crucial factor that has been investigated. Some studies have compared conditions in which pattern repetitions occur with strict isochronous timing (as was the case in the present study) to those in which the intervals between stimuli are jittered. While repetition detection remains robust under both conditions, the temporal regularity of the sequence can advance the onset or enhance the amplitude of the early positive ERP components (Costa-Faidella et al., 2011a; Ringer et al., 2023). The stronger RP response in isochronous stimuli suggests that temporal predictability enhances adaptation. When timing is uncertain, RP decreases, indicating that sensory memory sensitivity depends on predictability (Costa-Faidella et al., 2011a; Ringer et al., 2023). Taken together, these findings suggest that RP reflects an early stage where bottom-up adaptation is shaped by top-down predictive processes. When attention and task demands increase, precision weighting increases, resulting in sharper deviant perception and stronger MMN responses. When uncertainty is high, top-down control weakens, adaptation increases, but change detection becomes more difficult. Thus, bottom-up adaptation and top-down prediction function as complementary systems in auditory change perception (Friston and Kiebel, 2009; Winkler et al., 2009; Todd et al., 2012).

### Frequency coding in auditory system

Sound intensity and frequency perception are intricately linked through a cascade of processes that begin with the mechanical transduction in the cochlea and extend to complex cortical processing mechanisms. At the periphery, sound intensity influences the amplitude and spread of the traveling wave along the basilar membrane, thereby modulating the spatial pattern of hair cell activation that underlies frequency encoding (Grondin, 2016; Schreiner and Malone, 2015). RP following acoustic frequency and intensity changes were observed in our study, but to intensity only following increases. Greater stimulus intensity also leads to the activation of a wider frequency range of hair cells because off-frequency activation increases with intensity. Furthermore, neurophysiological studies in animal models have demonstrated that as sound intensity increases, the best frequency (BF) that elicits the maximum neuronal response can shift bidirectionally; neurons with low characteristic frequencies may shift their BF downward, while those with high characteristic frequencies shift upward, suggesting an expansion of frequency representation at extreme ends of the spectrum (Tao et al., 2017). In the present study, it is therefore possible that a prerequisite for RP to occur is the activation of additional neurons responsive to newly stimulated frequencies.

Active processes within the cochlea, driven by outer hair cells, provide non-linear amplification that sharpens frequency selectivity at low intensities while compressing dynamic range at higher volumes (Lesica, 2018). These peripheral events are mirrored by adaptive changes in the firing patterns of auditory nerve fibers, which transmit frequency-specific information via mechanisms such as phase locking (the ability of neurons to fire at a particular phase of the sound wave) and rate coding (Soland, 2022; Oxenham, 2018).

Central auditory structures, including the primary auditory cortex, exhibit a dynamic mapping of frequency that is modulated by sound intensity, as evidenced by intensity-dependent shifts in best frequency and expansion of cortical activation with increased loudness (Tao et al., 2017; Uppenkamp and Röhl, 2014). These cortical adaptations may serve to preserve robust pitch perception even in the face of variations in acoustic energy, thereby maintaining behavioral frequency discrimination despite changes in overall loudness (Schreiner and Malone, 2015; Oxenham, 2018).

Frequency information is neurally preserved in the IC and MGB, supporting the initial stages of SSA. Subcortical structures respond more stably to deviant frequencies and adapt more rapidly to standard stimuli. The auditory system integrates information from both the place and temporal domains to ensure accurate frequency perception despite variations in sound intensity (Costa-Faidella et al., 2011a; Monson et al., 2014). Increases in sound intensity lead to greater activation across auditory cortical regions such as Heschl's gyrus and the planum temporale, with these changes reflecting both the percept of loudness and alterations in spectral representation (Uppenkamp and Röhl, 2014). SSA becomes stronger and more frequency-selective at the cortical level, decreasing the response to repeated standard stimuli while preserving the response to deviant stimuli from suppression. SSA is the basis for the formation of a feature-specific memory trace at the cortical level (Costa-Faidella et al., 2011a; Ulanovsky et al., 2004).

### Intensity coding in auditory system

Intensity coding utilizes mechanical compression and neural firing rate in the peripheral system, while top-down effects are minimal. Sound intensity exerts a powerful influence on frequency perception by altering the mechanical response of the cochlea, modulating the firing properties of auditory nerve fibers, and dynamically shaping cortical tonotopic maps. At low intensities, the auditory system benefits from sharp frequency tuning facilitated by OHC amplification, which preserves the spatial precision of the neural code. However, as intensity increases, the non-linear characteristics of the cochlea lead to broader activation patterns that, while enhancing the overall loudness of the sound, may compromise fine frequency discrimination through a spreading of excitation (Grondin, 2016; Schreiner and Malone, 2015), which would propagate upwards through all stages of the central auditory pathway.

Animal studies further demonstrate that intensity-dependent shifts in best frequency in auditory cortex represent an adaptive mechanism that may expand the frequency range available for processing at higher sound levels, thereby compensating for potential losses in spectral resolution (Tao et al., 2017). As well as our earlier postulation about off-frequency stimulation increasing with stimulus intensity, it is possible that RP following intensity changes could have its basis in some of these other changes in neural reactivity, tonotopic representation, and/or sharpness of frequency responsivity caused by changing intensity. In the central system, both activation volume and percent signal change in the AC increase linearly in response to an increase in intensity. Expectations generated by the A1, belt, and frontal systems generate top-down predictions (Röhl and Uppenkamp, 2012).

### Long accumulation of RP

Previous studies of RP have typically used sequences of up to around 14 stimuli, and found greater RP with increasing number (Baldeweg, 2007; Costa-Faidella et al., 2011a). Our findings on the time course of RP do support this number as an optimal way of detecting RP overall, as the rate of increase slowed after this number. However, there may also be merits in establishing the plateau that RP eventually reaches, such as fully quantifying its extent. Furthermore, comparing the rate of formation of RP (distinctly from its magnitude) over stimuli might reveal important differences, such as between age groups, attentional states, or those with clinical disorders vs. control groups.

### Potential implications for clinical conditions

MMN is widely studied as a correlate of various mental health (Valt et al., 2023), age-related and neurodegenerative (Näätänen et al., 2011), neurodiversity (da Silva Mayerle et al., 2023) and sensory perceptual conditions (Yukhnovich et al., 2024b; Sendesen et al., 2021). Persistent auditory memory trace formation has been argued to be a key mechanism of tinnitus and chronic pain, for instance (De Ridder et al., 2011). Furthermore, P2 following acoustic stimuli, which overlaps strongly with RP and may even result from RP, has been shown to be increased in magnitude in people with hyperacusis, which in turn is at least part of the basis for increased MMN amplitudes in this group (as subtracting a more positive waveform results in a more negative one; Yukhnovich et al., 2024a). Lack of habituation to repetitive stimulation could be a symptom of autism, sometimes leading on the one hand to discomfort, and on the other hand to self-stimulation. With these observations and interrelationships in mind, RP might offer insights into a variety of clinical conditions, and form part of the toolkit for objectively demonstrating sensory processing abnormalities. It remains to be established whether RP following intensity changes may offer unique additional insights in these contexts.

### Limitations of the study

One limitation of the study is the assumption that a 12 dB change in intensity is equally salient as a 1 kHz change in frequency. Also, not making a power analyses for study example is the other limitations of the study. These were based on informal pilot work of our groups and also the previous studies (Recasens et al., 2015; Cacciaglia et al., 2019) showed that RP response can be observed with even a small number of sample size.

In this work, we studied adaptive processes occurring over timescales of tens of seconds, but did not examine changes in those adaptive processes occurring over longer timescales of tens of minutes, which would be an interesting angle for future work. Also, the source localization of RP to intensity could be interesting, in case it differs from frequency, but magnetoencephalography (MEG) might be the better modality to do this, given its greater spatial localization accuracy. Furthermore, we did not examine how frequency and intensity changes interact in eliciting RP, which represents an interesting direction for future research. RP with the stimuli of additional intensity levels (beyond 65 dB and 77 dB) is worthy of investigation in future works.

### Conclusions

Repetition positivity (RP) progressively occurs following increases in stimulus intensity, but not necessarily decreases. The relative lack of RP following intensity decreases suggests against it being a generic and ubiquitous marker of sensory memory trace strength or prediction precision, and may mean that its occurrence requires the activation of previously unstimulated neuronal populations. RP elicited in the context of intensity increases is qualitatively similar to RP that follows frequency changes, which have previously been studied, but appears smaller in amplitude and to reach saturation point after a smaller number of successive stimuli. If RP becomes a more staple part of the cognitive and sensory neuroscience toolkit, it will remain to see whether intensity-related RP may offer additional unique insights.

## Data Availability

The datasets presented in this study can be found in online repositories. The names of the repository/repositories and accession number(s) can be found at: https://figshare.com/s/14a1691e82d6b91b483c.

## References

[B1] Bader M. Schröger E. Grimm S. (2017). How regularity representations of short sound patterns that are based on relative or absolute pitch information establish over time: an EEG study. PLoS ONE 12:e0176981. doi: 10.1371/journal.pone.0176981 28472146 PMC5417614

[B2] Baldeweg T. (2006). Repetition effects to sounds: evidence for predictive coding in the auditory system. Trends Cogn. Sci. 10, 93–94. doi: 10.1016/j.tics.2006.01.010 16460994

[B3] Baldeweg T. (2007). ERP repetition effects and mismatch negativity generation: a predictive coding perspective. J. Psychophysiol. 21, 204–213. doi: 10.1027/0269-8803.21.34.204

[B4] Behler O. Uppenkamp S. (2016). The representation of level and loudness in the central auditory system for unilateral stimulation. Neuroimage 139, 176–188. doi: 10.1016/j.neuroimage.2016.06.025 27318216

[B5] Bendixen A. SanMiguel I. Schröger E. (2012). Early electrophysiological indicators for predictive processing in audition: a review. Int. J. Psychophysiol. 83, 120–131. doi: 10.1016/j.ijpsycho.2011.08.003 21867734

[B6] Cacciaglia R. Costa-Faidella J. Zarnowiec K. Grimm S. Escera C. (2019). Auditory predictions shape the neural responses to stimulus repetition and sensory change. Neuroimage 186, 200–210. doi: 10.1016/j.neuroimage.2018.11.007 30414982

[B7] Carbajal G. V. Malmierca M. S. (2018). The neuronal basis of predictive coding along the auditory pathway: from the subcortical roots to cortical deviance detection. Trends Hear. 22. doi: 10.1177/2331216518784822 30022729 PMC6053868

[B8] Cooper R. J. Atkinson R. J. Clark R. A. Michie P. T. (2013). Event-related potentials reveal modelling of auditory repetition in the brain. Int. J. Psychophysiol. 88, 74–81. doi: 10.1016/j.ijpsycho.2013.02.003 23454030

[B9] Costa-Faidella J. Baldeweg T. Grimm S. Escera C. (2011a). Interactions between “what” and “when” in the auditory system: temporal predictability enhances repetition suppression. J. Neurosci. 31, 18590–18597. doi: 10.1523/JNEUROSCI.2599-11.2011 22171057 PMC6623902

[B10] Costa-Faidella J. Grimm S. Slabu L. Díaz-Santaella F. Escera C. (2011b). Multiple time scales of adaptation in the auditory system as revealed by human evoked potentials. Psychophysiology 48, 774–783. doi: 10.1111/j.1469-8986.2010.01144.x 20946129

[B11] da Silva Mayerle M. C. C. Riesgo R. Gregory L. Borges V. M. S. Sleifer P. (2023). Mismatch negativity in children and adolescents with autism spectrum disorder. Int. Arch. Otorhinolaryngol. 27, e218–e25. doi: 10.1055/s-0043-1768209 37125353 PMC10147454

[B12] Davis K. A. (2005). Spectral processing in the inferior colliculus. Int. Rev. Neurobiol. 70, 169–205. doi: 10.1016/S0074-7742(05)70006-4 16472635

[B13] De Ridder D. Elgoyhen A. B. Romo R. Langguth B. (2011). Phantom percepts: tinnitus and pain as persisting aversive memory networks. Proc. Nat. Acad. Sci. U.S.A. 108, 8075–8080. doi: 10.1073/pnas.1018466108 21502503 PMC3100980

[B14] Delorme A. Makeig S. (2004). EEGLAB an open source toolbox for analysis of single-trial EEG dynamics including independent component analysis. J. Neurosci. Methods 134, 9–21. doi: 10.1016/j.jneumeth.2003.10.009 15102499

[B15] Friston K. Kiebel S. (2009). Predictive coding under the free-energy principle. Philos. Trans. R. Soc. B Biol. Sci. 364, 1211–1221. doi: 10.1098/rstb.2008.0300 19528002 PMC2666703

[B16] Garrido M. I. Kilner J. M. Kiebel S. J. Stephan K. E. Baldeweg T. Friston K. J. . (2009). Repetition suppression and plasticity in the human brain. Neuroimage 48, 269–279. doi: 10.1016/j.neuroimage.2009.06.034 19540921 PMC2821573

[B17] Grondin S. (2016). Psychology of Perception. Switzerland: Springer. doi: 10.1007/978-3-319-31791-5

[B18] Grotheer M. Kovács G. (2016). Can predictive coding explain repetition suppression? Cortex 80, 113–124. doi: 10.1016/j.cortex.2015.11.027 26861559

[B19] Haenschel C. Vernon D. J. Dwivedi P. Gruzelier J. H. Baldeweg T. (2005). Event-related brain potential correlates of human auditory sensory memory-trace formation. J. Neurosci. 25, 10494–10501. doi: 10.1523/JNEUROSCI.1227-05.2005 16280587 PMC6725828

[B20] Kohrman D. C. Borges B. C. Cassinotti L. R. Ji L. Corfas G. (2021). Axon–glia interactions in the ascending auditory system. Dev. Neurobiol. 81, 546–567. doi: 10.1002/dneu.22813 33561889 PMC9004231

[B21] Kumar S. Sedley W. Nourski K. V. Kawasaki H. Oya H. Patterson R. D. . (2011). Predictive coding and pitch processing in the auditory cortex. J. Cogn. Neurosci. 23, 3084–3094. doi: 10.1162/jocn_a_00021 21452943 PMC3821983

[B22] Lesica N. A. (2018). Why do hearing aids fail to restore normal auditory perception? Trends Neurosci. 41, 174–185. doi: 10.1016/j.tins.2018.01.008 29449017 PMC7116430

[B23] Monson B. B. Hunter E. J. Lotto A. J. Story B. H. (2014). The perceptual significance of high-frequency energy in the human voice. Front. Psychol. 5:587. doi: 10.3389/fpsyg.2014.00587 24982643 PMC4059169

[B24] Morrison J. A. Farzan F. Fremouw T. Sayegh R. Covey E. Faure P. A. . (2014). Organization and trade-off of spectro-temporal tuning properties of duration-tuned neurons in the mammalian inferior colliculus. J. Neurophysiol. 111, 2047–2060. doi: 10.1152/jn.00850.2013 24572091 PMC4044344

[B25] Näätänen R. (1995). The mismatch negativity: a powerful tool for cognitive neuroscience. Ear Hear. 16, 6–18. doi: 10.1097/00003446-199502000-00002 7774770

[B26] Näätänen R. Kujala T. Kreegipuu K. Carlson S. Escera C. Baldeweg T. . (2011). The mismatch negativity: an index of cognitive decline in neuropsychiatric and neurological diseases and in ageing. Brain 134, 3435–3453. doi: 10.1093/brain/awr064 21624926

[B27] Näätänen R. Paavilainen P. Titinen H. Jiang D. Alho K. (1993). Attention and mismatch negativity. Psychophysiology 30, 436–450. doi: 10.1111/j.1469-8986.1993.tb02067.x 8416070

[B28] Näätänen R. Picton T. (1987). The N1 wave of the human electric and magnetic response to sound: a review and an analysis of the component structure. Psychophysiology 24, 375–425. doi: 10.1111/j.1469-8986.1987.tb00311.x 3615753

[B29] Nelken I. Ulanovsky N. (2007). Mismatch negativity and stimulus-specific adaptation in animal models. J. Psychophysiol. 21, 214–223. doi: 10.1027/0269-8803.21.34.214

[B30] Norena A. Cransac H. Chery-Croze S. (1999). Towards an objectification by classification of tinnitus. Clin. Neurophysiol. 110, 666–675. doi: 10.1016/S1388-2457(98)00034-0 10378736

[B31] O'Reilly J. A. (2021). Can intensity modulation of the auditory response explain intensity-decrement mismatch negativity? Neurosci. Lett. 764, 136199. doi: 10.1016/j.neulet.2021.136199 34461160

[B32] Oxenham A. J. (2018). How we hear: the perception and neural coding of sound. Annu. Rev. Psychol. 69, 27–50. doi: 10.1146/annurev-psych-122216-011635 29035691 PMC5819010

[B33] Recasens M. Leung S. Grimm S. Nowak R. Escera C. (2015). Repetition suppression and repetition enhancement underlie auditory memory-trace formation in the human brain: an MEG study. Neuroimage 108, 75–86. doi: 10.1016/j.neuroimage.2014.12.031 25528656

[B34] Ringer H. Schröger E. Grimm S. (2023). Neural signatures of automatic repetition detection in temporally regular and jittered acoustic sequences. PLoS ONE 18:e0284836. doi: 10.1371/journal.pone.0284836 37948467 PMC10637696

[B35] Röhl M. Uppenkamp S. (2012). Neural coding of sound intensity and loudness in the human auditory system. J. Assoc. Res. Otolaryngol. 13, 369–379. doi: 10.1007/s10162-012-0315-6 22354617 PMC3346895

[B36] Schreiner C. E. Malone B. J. (2015). Representation of loudness in the auditory cortex. Handb. Clin. Neurol. 129, 73–84. doi: 10.1016/B978-0-444-62630-1.00004-4 25726263

[B37] Sendesen E. Erbil N. Türkyilmaz M. D. (2021). The mismatch negativity responses of individuals with tinnitus with normal extended high-frequency hearing—is it possible to use mismatch negativity in the evaluation of tinnitus? Euro. Arch. Oto-Rhino-Laryngol. 2021, 1–10. doi: 10.1007/s00405-021-07097-6 34564749

[B38] Soland K. (2022). Does loudness represent sound intensity? Synthese 200:100. doi: 10.1007/s11229-022-03665-3

[B39] Tao C. Zhang G. Zhou C. Wang L. Yan S. Zhou Y. . (2017). Bidirectional shifting effects of the sound intensity on the best frequency in the rat auditory cortex. Sci. Rep. 7:44493. doi: 10.1038/srep44493 28290533 PMC5349577

[B40] Todd J. Michie P. T. Schall U. Ward P. B. Catts S. V. (2012). Mismatch negativity (MMN) reduction in schizophrenia—Impaired prediction-error generation, estimation or salience? Int. J. Psychophysiol. 83, 222–231. doi: 10.1016/j.ijpsycho.2011.10.003 22020271

[B41] Ulanovsky N. Las L. Farkas D. Nelken I. (2004). Multiple time scales of adaptation in auditory cortex neurons. J. Neurosci. 24, 10440–10453. doi: 10.1523/JNEUROSCI.1905-04.2004 15548659 PMC6730303

[B42] Uppenkamp S. Röhl M. (2014). Human auditory neuroimaging of intensity and loudness. Hear. Res. 307, 65–73. doi: 10.1016/j.heares.2013.08.005 23973563

[B43] Uppenkamp S. Rohl M. (eds.) (2011). “Neural correlates of loudness perception in human auditory cortex using functional MR imaging,” in INTER-NOISE and NOISE-CON Congress and Conference Proceedings (Institute of Noise Control Engineering).

[B44] Valt C. Quarto T. Tavella A. Romanelli F. Fazio L. Arcara G. . (2023). Reduced magnetic mismatch negativity: a shared deficit in psychosis and related risk. Psychol. Med. 53, 6037–6045. doi: 10.1017/S003329172200321X 36321391

[B45] Winkler I. Denham S. L. Nelken I. (2009). Modeling the auditory scene: predictive regularity representations and perceptual objects. Trends Cogn. Sci. 13, 532–540. doi: 10.1016/j.tics.2009.09.003 19828357

[B46] Ylinen S. Huotilainen M. (2007). Is there a direct neural correlate for memory-trace formation in audition? Neuroreport 18, 1281–1284. doi: 10.1097/WNR.0b013e32826fb38a 17632283

[B47] Yukhnovich E. A. Alter K. Sedley W. (2023). Nuances in intensity deviant asymmetric responses as a biomarker for tinnitus. PLoS ONE 18:e0289062. doi: 10.1371/journal.pone.0289062 37549154 PMC10406247

[B48] Yukhnovich E. A. Alter K. Sedley W. (2024a). Distinct profiles of tinnitus and hyperacusis in intensity deviant responses and auditory evoked potentials. bioRxiv. doi: 10.1101/2024.01.02.573726

[B49] Yukhnovich E. A. Alter K. Sedley W. (2024b). What do mismatch negativity (MMN) responses tell us about tinnitus? J. Assoc. Res. Otolaryngol. 2024, 1–15. doi: 10.1007/s10162-024-00970-1 39681798 PMC11861849

